# The effect of electronic medical record adoption on outcomes in US hospitals

**DOI:** 10.1186/1472-6963-13-39

**Published:** 2013-02-01

**Authors:** Jinhyung Lee, Yong-Fang Kuo, James S Goodwin

**Affiliations:** 1 Department of Internal Medicine, University of Texas Medical Branch, Galveston, TX, 77555, USA

**Keywords:** Electronic medical record (EMR), Length of stay, Rehospitalization rates, Diagnosis related groups (DRG)

## Abstract

**Background:**

The electronic medical record (EMR) is one of the most promising components of health information technology. However, the overall impact of EMR adoption on outcomes at US hospitals remains unknown. This study examined the relationship between basic EMR adoption and 30-day rehospitalization, 30-day mortality, inpatient mortality and length of stay.

**Methods:**

Our overall approach was to compare outcomes for the two years before and two years after the year of EMR adoption, at 708 acute-care hospitals in the US from 2000 to 2007. We looked at the effect of EMR on outcomes using two methods. First, we compared the outcomes by quarter for the period before and after EMR adoption among hospitals that adopted EMR. Second, we compared hospitals that adopted EMR to those that did not, before and after EMR adoption, using a generalized linear model.

**Results:**

Hospitals adopting EMR experienced 0.11 (95% CI: -0.218 to −0.002) days’ shorter length of stay and 0.182 percent lower 30-day mortality, but a 0.19 (95% CI: 0.0006 to 0.0033) percent increase in 30-day rehospitalization in the two years after EMR adoption. The association of EMR adoption with outcomes also varied by type of admission (medical vs. surgical).

**Conclusions:**

Previous studies using observational data from large samples of hospitals have produced conflicting results. However, using different methods, we found a small but statistically significant association of EMR adoption with outcomes of hospitalization.

## Background

The electronic medical record (EMR) is designed to improve communication among providers within and between organizations by automating the collection, use and storage of patient information. Moreover, EMR may facilitate guideline compliance and decision support [1]. Numerous single site studies at academic hospitals provide evidence that specific functions of the EMR, such as clinical decision support or computerized physician order entry, may improve quality by reducing errors [2-4]. Other studies using large samples of hospitals have found evidence that overall spending on health information technology (IT) is associated with improved patient safety, higher quality of care and reduced costs [5-9]. The Institute of Medicine (IOM) has encouraged adopting EMR to reduce medical errors, and the American Recovery and Reinvestment Act (ARRA) of 2009 established financial incentives for hospitals to promote the adoption and meaningful use of health IT.

Despite efforts to encourage the adoption of EMR, the impact of EMR on outcomes at US hospitals remains unknown. In particular, the effects of EMR on 30-day rehospitalization, 30-day mortality, inpatient mortality and length of stay have not been well characterized at community hospitals. Understanding how EMR affects hospital care outcomes can help policy makers promote its effective use.

This study examines the relationship between basic EMR adoption and 30-day rehospitalization, 30-day mortality, inpatient mortality and length of stay at 708 acute-care hospitals in the US. We used the Healthcare Information and Management Systems Society (HIMSS) data merged to 5% Medicare data from 2000 to 2007. We used two approaches to control for time-varying unobservable events that may have occurred concurrently with EMR adoption. First, we compared the slope of change in outcomes before and after EMR adoption among hospitals that adopted EMR. Second, we compared difference in outcomes before and after EMR adoption between EMR and non-EMR adopted hospitals. Further, we examined how the effect of EMR on outcomes varied by type of admission and by hospital characteristics.

## Methods

### Source of data

There were four primary sources of data: Healthcare Information and Management Systems Society (HIMSS) data, Medicare enrollment files, Provider of Service files and the 5% Medicare Provider Analysis and Review (MEDPAR) data from 2000 to 2007. HIMSS provides information on IT applications in approximately 3,000 hospitals in the US. The HIMSS sample comes from the American Hospital Association (AHA) survey and includes nearly all general hospitals. Medicare enrollment files include patient characteristics such as age, sex, race and Medicare and health maintenance organization (HMO) enrollment information. The Provider of Service file gives provider number, name, and address and characteristics of participating institutional providers. The MEDPAR file contains data from claims for services provided to beneficiaries admitted to Medicare certified inpatient hospitals and skilled nursing facilities. The study protocol was approved by the institutional review board at the University of Texas Medical Branch (IRB#: 09–054).

### Establishment of the study cohort

We identified around 2,600 unique and 20,565 pooled acute-care hospitals with at least 100 beds in HIMSS data from 2000 to 2007. With these data, we kept only hospitals which could be observed during all eight years from 2000 to 2007 and which had a Medicare provider number. The remaining sample was 1,710 unique and 13,680 pooled hospitals from 2000 to 2007. We excluded the 1,002 hospitals which had adopted some component of a basic EMR before 2002. Thus, the final sample comprised 708 acute care hospitals: 425 that adopted basic EMR in 2002–2005 and 283 that did not adopt basic EMR during this period. We used HIMSS data to identify the year of implementation of a basic EMR, defined as a computerized patient record supported by a clinical data repository and providing clinical decision-support capabilities [10,11].

We explored four outcomes: 30-day rehospitalization, 30-day mortality, inpatient mortality and length of stay. The sample included beneficiaries age 66 or older not enrolled in HMOs and with both Medicare Parts A and B for the entire 12 months before admission. For the hospitals that adopted EMR, admissions before EMR adoption accounted for 38% of all admissions in the sample, while those after EMR adoption accounted 41%. Another 21% of admissions occurred within the EMR adoption year.

#### 30-day rehospitalization

The accumulation of claims from a beneficiary’s date of hospital admission until discharge represents one stay in the MEDPAR file. We included only stays shorter than 365 days and those from which the patient was discharged alive. We defined the rate of rehospitalization by dividing the number of patients discharged from an acute hospital and readmitted to any acute hospital within 30 days by the total number of patients discharged alive. For enrollees with multiple admissions, we randomly selected one admission per year. Our final sample size was 237,081.

#### In-patient mortality and 30-day mortality

We included only stays shorter than 365 days and all patients, including those who died in the hospital or were discharged alive. We defined the rate of in-patient mortality by dividing the number of patients who died during their stay by the total number of patients admitted. Also, we defined the rate of 30-day mortality by dividing the number of patients who died within 30 days after admission by the total number of patients admitted. Our final sample size was 403,566.

#### Length of stay

We included only stays shorter than 365 days and those from which the patient was discharged alive. To reduce skew, we excluded admissions in which length of stay was more than three standard deviations above the mean. Our final sample was 360,105 admissions.

### Measures

Medicare enrollment files were used to categorize patients according to age, sex and race (White, Black or Other). Information on discharge DRG and DRG weight were obtained from the MEDPAR files and Center for Medicare and Medicaid Service (CMS), respectively. Elixhauser co morbidity [12] measures were generated for the 12 months before admission using inpatient and physician claims from MEDPAR, Outpatient Statistical Analysis files, and Carrier files.

Hospital characteristics were obtained from the Provider of Service files, including teaching status, number of beds and ownership type. Teaching status was categorized as none and any. Hospital size was grouped into quartiles based on the number of beds (100–170; 171–260; 261–390; and over 390) and ownership type of hospitals was grouped as not-for-profit, for-profit and public.

### Methodology and statistical analysis

Our approach was to compare outcomes for the two years before and two years after the year of EMR adoption. Four hundred twenty-five hospitals adopted EMR from 2002 to 2005 (159 hospitals in 2002, 77 in 2003, 46 in 2004 and 143 in 2005). Hospitals were assigned a “-2” value at two years before their EMR adoption, “-1” for one year before EMR adoption, “0” for the year of EMR adoption, “1” for the year after EMR adoption and “2” for the period two years after EMR adoption. For example, the 159 hospitals that adopted EMR in 2002 were assigned −2, -1, 0, 1 and 2 in the years 2000, 2001, 2002, 2003 and 2004, respectively. We then stacked the data by EMR adoption year.

We examined the effect of EMR on outcomes two different ways. First, we compared the slope of change in outcomes, by quarter, before and after EMR adoption among hospitals that adopted EMR by using piecewise linear regression. We can capture any cut point if there is any slope change in outcomes.

Second, we compared difference in outcomes before and after EMR adoption between EMR and non-EMR adopted hospitals. In these analyses, the study year “0” for hospitals without EMR adoption was assigned randomly to match the distribution for the year of adoption for hospitals the adopted EMR (106 in 2002, 51 in 2003, 30 in 2004 and 96 in 2005).

We used generalized linear models (GLM) and adjusted the four outcomes (30-day rehospitalization, 30-day mortality, inpatient mortality and length of stay) by patient characteristics (gender, age and race), disease characteristics (DRG weight and comorbidities) and year. For 30-day rehospitalization, 30-day mortality and inpatient mortality, GLM with binomial distribution and logit link function was used. For length of stay, GLM with gamma^a^ distribution and log link function were used. All of these models accounted for the clustering of patients within hospitals. STATA statistical software, version 11.1 (STATA Corp., College Station, TX) was used for all analyses.

## Results

The study cohort consisted of patients admitted to 425 hospitals that adopted EMR during the period 2002 to 2005, and 283 hospitals that did not adopt EMR over the eight years from 2000 to 2007. Table 1 presents patient and disease characteristics in the EMR and non-EMR hospitals. We found that patients were slightly more likely to be male, be Black and have higher DRG weight in EMR hospitals than in non-EMR hospitals. However, age and comorbidity were higher in non-EMR hospitals than in EMR hospitals. Also, because of the large sample size, all other variables were statistically different between EMR and non-EMR hospitals (all p < 0.01). Thus, it is more important to focus on the magnitude of any differences rather than their level of statistical significance.

**Table 1:** Characteristics of patients and disease two years before and after EMR adoption, based on 425 EMR-adopting hospitals

**Variable**		**EMR hospital**	**Non EMR hospital**	**P-value**
		**Mean (SD)**	**Mean (SD)**	
Number of Sample		360,105	313,088	
Male		41.49%	41.00%	< 0.01
Race	White	86.60%	87.23%	< 0.01
	Black	9.47%	8.59%	< 0.01
	Other	3.93%	4.18%	< 0.01
Age		78.35 (7.67)	78.63(7.75)	< 0.01
DRG weight		1.58 (1.33)	1.53 (1.31)	< 0.01
Comorbidity		2.84 (1.74)	3.42 (2.59)	< 0.01

*EMR*, electronic medical records; *SD*, standard deviation; *DRG*, diagnosis related group.

Comorbidity: Elixhauser comorbidity were generated for the 12 months before admission using inpatient and physician claims from MEDPAR, Outpatient Statistical Analysis Files, and Carrier files.

Included only patients not enrolled in HMO and with both Medicare Parts A and B for the entire 12 months before admission. Excluded discharged to other acute care hospitals.

**Figure 1:**
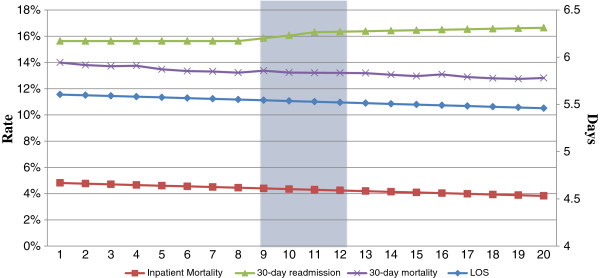
Outcomes in EMR adopted group two years before and two years after EMR adoption year.

First, we examined changes in outcomes before (8 quarters) and after (12 quarters) EMR adoption in the 425 hospitals that adopted EMR during the period 2002 to 2005 (Figure 1). For each hospital, the year of EMR adoption was designated as year 0. We used piecewise linear regression to assess if there was a change in slope in any of the outcomes over the entire 20 quarters. For 30-day rehospitalization rate and 30-day mortality, there was a significant change in slope over time (p < 0.001 for each). In both cases, the cutpoint was in the first quarter of the year of EMR implementation. As shown in Table 2, the odds of 30-day rehospitalization was stable in the eight quarters prior to EMR adoption and increased at 0.00037 odds per quarter after. Similarly, the odds of 30-day mortality was stable in the eight quarters prior to EMR adoption and decreased at 0.00062 odds per quarter after. There were no significant changes in slope for length of stay or inpatient mortality over the 20 quarters. We used a similar approach with piecewise linear regression to determine the temporal changes in outcomes in the 283 hospitals that did not adopt EMR over the 2000–2007 period. For length of stay, inpatient mortality and rehospitalization rate, there were underlying temporal trends noted in the hospitals that did not adopt EMR within the 2002–2005 window. To address the concern of underline temporal trend, we conducted further analyses focusing on the differences in changes in outcomes over time between EMR adopted and non-EMR adopted hospitals.

**Table 2:** Slope difference before and after EMR adoption, based on 425 EMR-adopting hospitals

	**Slope before EMR**	**Slope after EMR**	**Slope difference**
30-day rehospitalization	1.00000	1.00037	0.00037
30-day mortality	1.00000	0.99938	−0.00062

Slope represents the odds ratio per quarter.

Slope difference was defined by subtracting the slope “after EMR” from the slope “before EMR.”

The “before EMR” slope includes 8 quarters before EMR adoption and the “after EMR” slope includes 12 quarters after EMR adoption.

All outcomes were adjusted by patient (gender, age and race) and disease characteristics (DRG weight and comorbidities).

Accordingly, we compared changes over time in the outcomes for hospitals that adopted EMR compared to those that did not. Once again, hospitals that adopted EMR during 2002–2005 were stacked so that the year of EMR adoption was year 0. We randomly assigned hospitals that did not adopt EMR to the same years and stacked them in similar fashion. We calculated the difference in each outcome between the EMR and non-EMR hospitals in the eight quarters before and the eight quarters after the year of EMR adoption (Table 3). For example, in Table 3, the rate of 30-day rehospitalization in EMR hospitals in the eight quarters prior to EMR adoption was 0.46 percent less than in non-EMR hospitals. In the two years after EMR adoption, this difference decreased to −0.26 percent. Thus, relative to non-EMR hospitals, patients in hospitals that adopted EMR had a 0.19 percent higher rate of rehospitalization in the two years after EMR adoption (p < 0.01). We also found that EMR was associated with shorter length of stay and lower 30-day mortality, even though the outcome changes associated with EMR adoption were small. Hospitals that adopted the EMR system experienced a 0.11 decrease in length of stay (P < 0.05) and 0.18 percent decrease in 30-day mortality (P < 0.01).

**Table 3:** Difference in outcomes for hospitals that adopted EMR (n = 425) vs. non-EMR (n = 283) hospitals, before and after the years of EMR adoption

	**30-day rehospitalization**				**30-day mortality**			
	**Before**		**After**		**Before**		**After**	
	**EMR**	**Non-EMR**	**EMR**	**Non-EMR**	**EMR**	**Non-EMR**	**EMR**	**Non-EMR**
Outcomes	15.64%	16.10%	16.49%	16.76%	13.58%	13.96%	12.94%	13.51%
(Std. Err)	(0.04)	(0.06)	(0.06)	(0.03)	(0.09)	(0.05)	(0.06)	(0.05)
Difference between EMR and non-EMR hospitals		−0.46%		−0.26%		−0.39%		−0.57%
(Std. Err)		(0.02)		(0.05)		(0.06)		(0.03)
Difference before and after				0.19% **				−0.18% **
(Std. Err)				(0.06)				(0.07)
	**Inpatient mortality**				**Length of stay**			
	**Before**		**After**		**Before**		**After**	
	**EMR**	**Non-EMR**	**EMR**	**Non-EMR**	**EMR**	**Non-EMR**	**EMR**	**Non-EMR**
Outcomes	4.69%	4.67%	4.10%	4.16%	5.58	5.92	5.49	5.93
(Std. Err)	(0.05)	(0.02)	(0.04)	(0.01)	(0.01)	(0.03)	(0.01)	(0.04)
Difference between EMR and non-EMR hospitals		0.01%		−0.05%		−0.33		−0.44
(Std. Err)		(0.03)		(0.02)		(0.03)		(0.01)
Difference before and after				−0.07%				−0.11 day *
(Std. Err)				(0.05)				(0.05)

Difference between EMR and non-EMR hospitals was calculated by subtracting the mean values of non-EMR adopted hospitals from the mean values of EMR adopted hospitals.

Difference before and after was calculated by subtracting the mean values in the eight quarters before the year of EMR adoption with the values in the eight quarters after the year of EMR adoption.

All outcomes were adjusted by patient (gender, age and race) and disease characteristics (DRG weight and comorbidities).

** p < 0.01, * p < 0.05.

However, there were no significant changes in inpatient mortality before and after EMR implementation. For inpatient mortality, length of stay may be an important factor because patients with critical conditions are more likely to both stay longer and die during hospitalization [13]. Thus, we also compared the inpatient mortality rate after adjusting length of stay with other variables mentioned above, but found no significant difference in outcomes between EMR and non-EMR adopted hospitals.

Moreover, we stratified DRGs into surgical and medical based on the DRG [14] and compared outcomes between EMR and non-EMR adopting hospitals (Table 4). We found that the effect of EMR on outcomes differed by DRG type. EMR reduced the inpatient morality rate in surgical DRGs, but it increased 30-day mortality. In medical DRGs, however, EMR increased length of stay and 30-day rehospitalization but reduced 30-day mortality.

**Table 4:** Difference between outcomes in hospitals that adopted EMR (n = 425) vs. non-EMR (n = 283) hospitals, before and after the years of EMR adoption by DRG type (surgical or medical) and hospital characteristics (for all DRGs)

**DRGs**		**Number of hospitals**	**30-day rehosp**		**30-day mortality**		**Inpatient mortality**		**Length of stay**	
			**Difference in outcome (Std. Err)**		**Difference in outcome (Std. Err)**		**Difference in outcome (Std. Err)**		**Difference in outcome (Std. Err)**	
Surgical DRGs			−0.05%		0.23% *		−0.24% **		−0.11 day	
			(0.09)		(0.08)		(0.06)		(0.13)	
Medical DRGs			0.37% **		−0.22% **		−0.01%		0.07 day**	
			(0.06)		(0.07)		(0.05)		(0.023)	
**Hospital characteristics**				**P-value**		**P-value**		**P-value**		**P-value**
Number of Bed	1st Quartile (Less than 170)		0.24 % **	0.71	0.06 %	0.17	0.08% *	0.16	0.04 day	0.42
		185	(0.08)		(0.08)		(0.03)		(0.07)	
	2nd Quartile (170–260)		0.32% **		−0.11 %		−0.03 %		−0.07 day	
		188	(0.06)		(0.00)		(0.05)		(0.05)	
	3rd Quartile (261–390)		0.26 % **		−0.14 **		−0.02%		−0.19 day	
		177	(0.06)		(0.04)		(0.04)		(0.13)	
	4th Quartile (Over 390)		0.33 % **		0.02		0.07%		- 0.07 day	
		158	(0.06)		(0.08)		(0.04)		(0.11)	
Teaching Status	Teaching		0.29 % **	0.99	−0.05 %	0.65	−0.004 %	0.55	- 0.01 day	0.20
		399	(0.05)		(0.05)		(0.06)		(0.08)	
	Non-Teaching		0.29 % **		−0.02 %		0.03%		−0.16 day *	
		309	(0.07)		(0.06)		(0.04)		(0.09)	
Ownership	For-profit		0.27 % **	0.02	−0.02 %	0.02	0.01%	0.95	−0.05 day	0.28
		551	(0.06)		(0.04)		(0.03)		(0.05)	
	Not-for-profit		0.07 %		−0.20 %		0.04 %		0.08 day	
			(0.08)		(0.16)		(0.11)		(0.14)	
	Public	55	0.42 % **		−0.09 %		0.03 %		−0.39 day	
		102	(0.11)		(0.09)		(0.06)		(0.33)	

Difference in outcomes was calculated by subtracting the mean values in the eight quarters before the year of EMR adoption with the values in the eight quarters after the year of EMR adoption.

All outcomes were adjusted by patient (gender, age and race) and disease characteristics (DRG weight and Comorbidities).

** p < 0.01, * p < 0.05.

We also compared outcomes of all DRGs across hospital characteristics including number of beds, teaching status and ownership (Table 4). Thus, each outcome was analyzed for four stratified sizes (by number of beds), two teaching states and three types of ownership. Across hospital characteristics, we found that the hospitals with large bed size, teaching and profit ownership status are more likely adopt EMR. However, we did not find any association between hospital characteristics and outcomes before and after EMR adoption (all p > 0.1), except for ownership and 30-day rehospitalization.

## Discussion

We found that EMR adoption was associated with a small but significant reduction in length of stay and 30-day mortality, as well as an increase in 30-day rehospitalization. The reduced length of stay associated with EMR suggests that EMR might allows faster physician ordering of tests, procedures, and medications, speed the process/scheduling of discharge, and reduce delays in the service ordering process. Also, through faster and accurate communication and coordination among providers, EMR may contribute to reduced length of stay [15].

However, shorter length of stay may increase the 30-day rehospitalization rate because patients with a critical condition may return if they are discharged early, a problem which EMR may catch. For example, Cram et al. examine demographics and outcomes of patients undergoing primary and revision total hip arthroplasty using Medicare Data. They found that length of stay decreased but the readmission rate increased [16]. Lin et al. used a nationwide population based dataset and found an increased adjusted odds ratio for 30-day readmission rate with shorter length of stay for patients with schizophrenia [17]. Moreover, Kuo and Goodwin used Medicare Data and found that reduced length of stay resulted in more readmissions within 30 days of discharge [18].

Reduced mortality may be a byproduct of better oversight. EMR can improve the continuum of care, alert physicians and nurses to when time-sensitive care processes are imminent and improve accurate communication to minimize complications from preventable errors [19].

Moreover, we found that EMR was associated with different DRGs across outcomes. However, the direction of outcome changes associated with EMR was not consistent across surgical and medical DRGs. Further, we did not find evidence of the effect of hospital characteristics on outcomes before and after EMR adoption because the different settings in hospitals may not capture the small outcome difference we observed.

These findings are comparable with most recent studies. Prior studies using observational data from large samples of hospitals have produced conflicting results. DesRoches et al. [20] used cross sectional data from 2,953 national hospitals and did not find any significant relationship between the existence of an EMR and 30-day mortality, length of stay and 30-day readmission rate. Furukawa et al. [21] used longitudinal data from 326 California hospitals and found that EMR was associated with longer length of stay and lower rates of in-hospital mortality. Amarasingham et al. [22] examined the cross sectional relationship between the level of automation and inpatient mortality in 41 Texas hospitals. They found that having an automation system was associated with fewer complications, lower mortality rates, and lower costs, but with no difference in length of stay. Compared to previous studies of hospitals throughout the US, our study results may be more representative because we used longitudinal data analysis of 708 acute care hospitals from 2000 to 2007 with different methods.

### Limitation

Small changes in outcomes associated with EMR might reflect the fact that the hospital may still be working to implement the system, because we focused on the short-term effects of EMR adoption during the first two years of implementation and did not consider the long-term effects of EMR use over time. This partial automation processes may delay the patient information transfer or induce workarounds, errors, and dissatisfaction from providers [23].

There may be unobserved confounding factors that might have impacted our results. For example, organizational and management strategies may be correlated with EMR adoption [24]. In particular, if the manager or CEO adopted more IT systems with EMR to improve their worst outcomes, then our estimated coefficient may be underestimated. Another concern is the physician’s perception of EMR use. EMR was considered to be adopted if the hospitals adopt EMR, not the physician. Thus, if physicians are resistant to using EMR after EMR adoption in hospitals, our estimate may be underestimated. Although we controlled for the characteristics of patients and hospitals, EMR adoption behavior may differ in some factors that we could not observe in our data (i.e. changes in reimbursement, policy, etc.). Thus, our estimates may remain biased.

We used the operational definition of EMR as the computerized patient record supported by a clinical data repository and clinical decision-support capabilities [10,11]. Our definition of EMR adoption is less restrictive than that used in other studies [25-27]. For example, Tucker and Miller [25] used HIMSS data but defined EMR adoption as complete adoption of four components including computerized practitioner order entry, clinical data repositories, clinical decision making support software, and systems designed to digitize physician documentation. Also, other studies have used a more restrictive definition of EMR [26,27] which included such IT systems as Nursing Documentation and Electronic Medication Administration Records. We used a less restrictive EMR definition because HIMSS data did not follow up on some IT systems (i.e., physician documents) adopted before 2005.

Also, we focused only on the availability of EMR. However, what is important is meaningful use, which is increasingly emphasized in federal policy. We did not have information on more relevant variables. Thus, future work needs to examine how EMR is effectively used.

## Conclusions

In summary, we found evidence for small but clinical significant changes in reduction of length of stay and 30-day mortality but an increase in 30-day rehospitalization with no changed in inpatient mortality with the introduction of a basic EMR in US hospitals.

## Endnote

^a^We selected gamma distribution after modified park test.

## Competing interest

The authors declare that they have no competing interests

## Authors’ contributions

JL was involved in research concept and design, collection and/or assembly of data, data analysis and interpretation and writing the article. YK was involved in research design, data interpretation and helped to draft the manuscript. JG was involved in research design, data interpretation and helped to write and review this work. All authors read and approved the final manuscript.

## Pre-publication history

The pre-publication history for this paper can be accessed here:

http://www.biomedcentral.com/1472-6963/13/39/prepub
